# Viral genome sequencing by random priming methods

**DOI:** 10.1186/1471-2164-9-5

**Published:** 2008-01-07

**Authors:** Appolinaire Djikeng, Rebecca Halpin, Ryan Kuzmickas, Jay DePasse, Jeremy Feldblyum, Naomi Sengamalay, Claudio Afonso, Xinsheng Zhang, Norman G Anderson, Elodie Ghedin, David J Spiro

**Affiliations:** 1 Viral Genomics Group, J. Craig Venter Institute, Rockville, MD 20850, USA; 2 Southeast Poultry Research Laboratory, Agricultural Research Service, US Department of Agriculture, Athens, GA 30605, USA; 3 Food Animal Health Research Program, Ohio Agricultural Research and Development Center, The Ohio State University, Wooster, OH 44691, USA; 4 Viral Defense Foundation, Kensington, MD 20891, USA; 5 Division of Infectious Diseases, University of Pittsburgh School of Medicine, Pittsburgh, PA 15261, USA

## Abstract

**Background:**

Most emerging health threats are of zoonotic origin. For the overwhelming majority, their causative agents are RNA viruses which include but are not limited to HIV, Influenza, SARS, Ebola, Dengue, and Hantavirus. Of increasing importance therefore is a better understanding of global viral diversity to enable better surveillance and prediction of pandemic threats; this will require rapid and flexible methods for complete viral genome sequencing.

**Results:**

We have adapted the SISPA methodology [1-3] to genome sequencing of RNA and DNA viruses. We have demonstrated the utility of the method on various types and sources of viruses, obtaining near complete genome sequence of viruses ranging in size from 3,000–15,000 kb with a median depth of coverage of 14.33. We used this technique to generate full viral genome sequence in the presence of host contaminants, using viral preparations from cell culture supernatant, allantoic fluid and fecal matter.

**Conclusion:**

The method described is of great utility in generating whole genome assemblies for viruses with little or no available sequence information, viruses from greatly divergent families, previously uncharacterized viruses, or to more fully describe mixed viral infections.

## Background

The emergence of highly pathogenic viral agents from zoonotic reservoirs has energized a wave of research into viral ecology, viral discovery [4-7] and a parallel drive to develop large datasets of complete viral genomes for the study of viral evolution and pandemic prediction [8,9]. Viral discovery has been aided by the development of sequence independent methodologies for the generation of genomic data [10]. The most prominent of these methodologies include representational difference analysis (RDA) and sequence independent single primer amplification (SISPA) with several variations. The SISPA method, first developed by Reyes and Kim [11], entails the directional ligation of an asymmetric primer at either end of a blunt-ended DNA molecule. Following several cycles of denaturation, annealing and amplification, minute amounts of the initial DNA are enriched and then cloned, sequenced and analyzed. Several modifications of the SISPA method have so far been implemented including random-PCR (rPCR) [12]. The rPCR method combines reverse transcription primed with an oligonucleotide made up of random hexamers tagged with a known sequence which is subsequently used as a primer-binding extension sequence. This initial modification was first used to construct a whole cDNA library from low amounts of viral RNA. A more recent modification, the DNAse-SISPA technique [1,2,5], includes steps to detect both RNA and DNA sequences. Combining sample filtration through a 0.22 micrometer column and a DNAse I digestion step led to the identification of viruses from clinical samples. The DNase-SISPA technique has been used for the detection of novel bovine and human viruses from screens of clinical samples [1,2,13]. Other groups have used the protocol for the characterization of common epitopes in enterovirus [14], for the identification of a novel human coronavirus [15] and for viral discovery in the plasma of HIV infected patients [16].

In addition to its utility for viral discovery and viral surveillance, the DNase-SISPA method has utility in obtaining full genome sequence from uncharacterized viral isolates or viral isolates from highly divergent families. In this study, we demonstrate the utility of the SISPA method and its use as a rapid and cost effective method for generating full genome coverage of a wide range of viral types from several sources.

## Results and discussion

### Optimization of the SISPA method for whole genome sequencing

**Figure 1:**
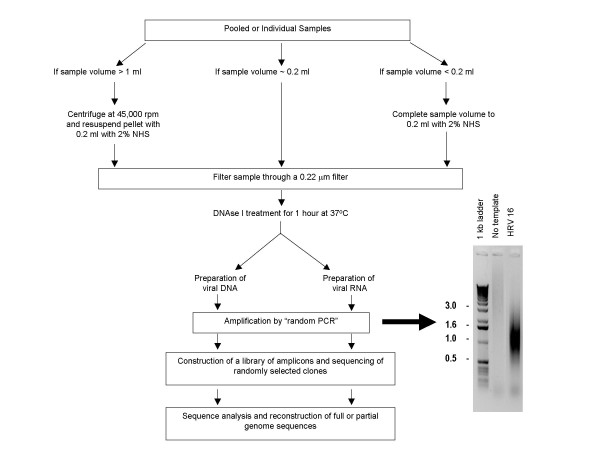
**Overview of the strategy**. Viral particles are separated from host contaminants using centrifugation and filtration. Viral particles are treated with DNAse I to remove contaminated nucleic acids. Random priming is used to generate 500–1000 bp amplicons which are size-selected and cloned. Colonies are picked and sequenced. Sequence is trimmed and assembled. Contigs are closed using sequence-specific primers.

**Figure 2:**
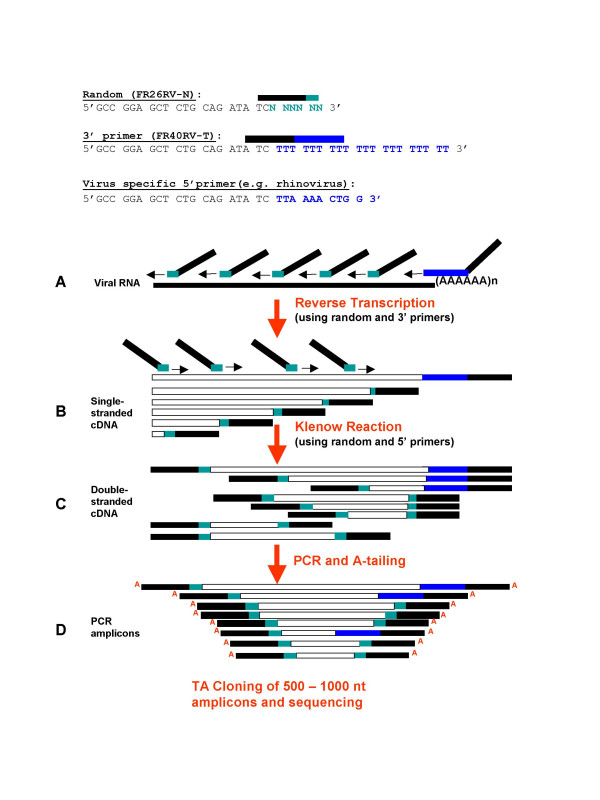
**Outline of the SISPA method**. A. Viral RNA is converted to cDNA using random-tagged and poly-A tagged primers (FR26RV-N and FR40RV-T). B. Second strand DNA is synthesized using Klenow exo-DNA polymerase, in the presence of random tagged and virus specific 5' end oligo primers. C. Double stranded DNA is amplified by PCR using the primer tag (FR20RV). D. Amplicons are separated by electrophoresis and products ranging from 500–1000 nucleotides are cloned into the TOPO vector. 96–288 colonies are picked, plasmid DNA is purified and the inserts are sequenced.

Given the success of earlier efforts for the identification of novel viral nucleic acids using SISPA, we sought to adapt and optimize this method as a general and cost effective technique for large scale *de novo *viral genome sequencing (Figures 1 and 2). An RNase treatment step was added to the SISPA protocol to reduce contaminating exogenous RNAs such as ribosomal RNAs. In the case of polyA-tailed viruses, we perform reverse transcription using a combination of random (FR26RV-N) and poly T tagged (FR40RV-T) primers in order to increase the coverage of the 3' end (Figure 2). Additionally, in order to capture 5' ends of viral RNA, a random hexamer primer tagged with a conserved sequence at the 5' end was added to the Klenow reaction (Figure 2 shows a 5' oligo specific for rhinoviruses).

### Viral genome assembly metrics

**Figure 3:**
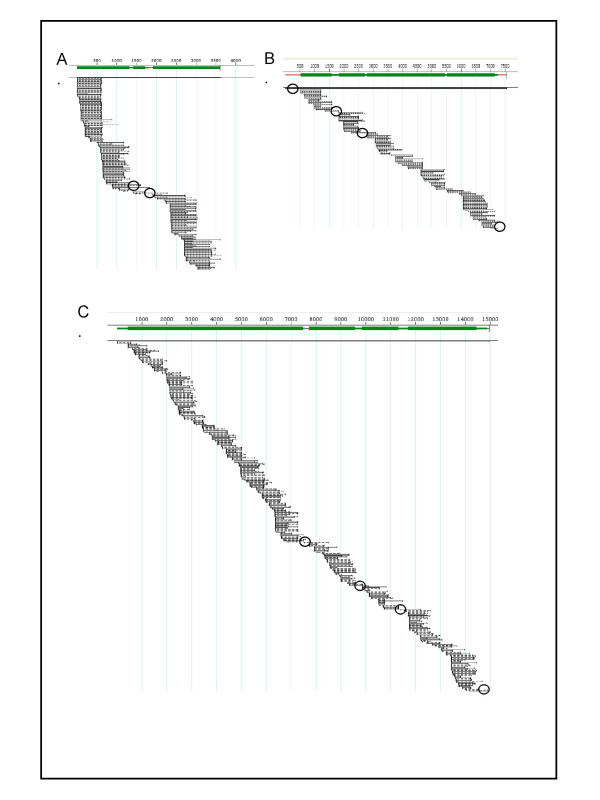
**Representative assemblies of viruses described in this study**. Images shown were generated using DNASTAR Seqman program. A. Enterobacteriophage MS2 (3569 bp). B. Human Rhinovirus 16 (7124 bp). C. Newcastle disease virus Lasota (15186 bp). All assemblies have been aligned with their reference genomes. Gaps and low coverage areas which require closure are circled.

We have successfully used the SISPA method on viral samples from different viral types. In this paper we discuss seven representative samples (Table 1). We have found that the method works consistently on dsDNA, ssDNA, ssRNA positive and ssRNA negative viruses. We have also found that the method can result in complete genome sequence of viruses ranging in size from 3,000–15,000 kb in a single experimental procedure. Figure 3 shows the sequence coverage obtained for three viruses: positive ssRNA phage MS2, positive ssRNA rhinovirus and negative ssRNA Newcastle disease virus (NDV).

**Table 1:** Viral isolates discussed in this study.

**Virus Name**	**Viral Type**	**Genome Size**	**Viral Particles**
Woodchuck hepatitis virus (WHV)	dsDNA	3308	n/a
Enterobacteriophage MS2 (MS2)	ssRNA positive	3569	10^8^
Enterobacteriophage M13 (M13)	ssDNA	6407	10^8^
Human Rhinovirus 16 (HRV16)	ssRNA positive	7124	n/a
Turkey Astrovirus (TA)	ssRNA positive	7355	n/a
Newcastle disease virus (NDV)	ssRNA negative	15186	n/a
Bacteriophage lambda (lambda)	dsDNA	48502	10^8^

**Figure 4:**
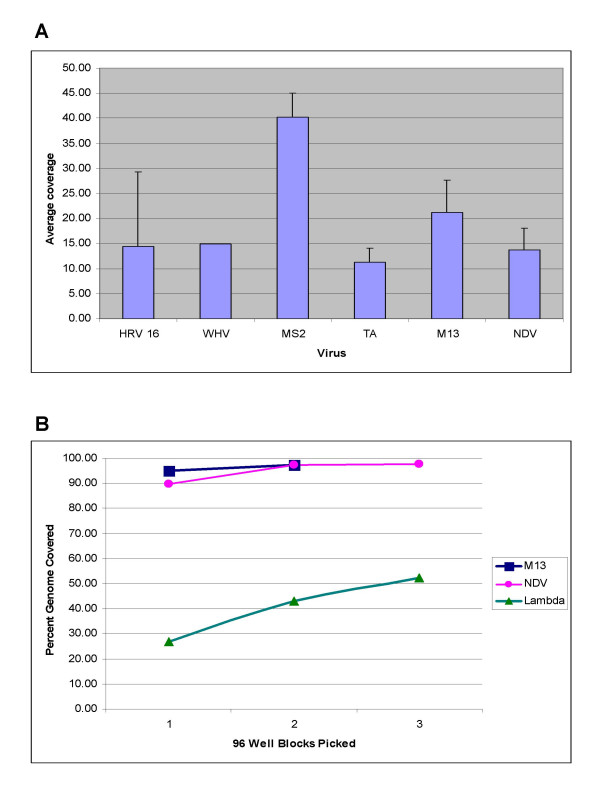
**A. Depth of coverage of viruses**. Depth of coverage statistics were generated for each contig (using the output of DNAStar Seqman program). Average coverage is the summed length of all sequence reads in a contig, including gaps divided by the contig length. The average and standard deviation for each virus was determined. **B. Correlation of genome coverage with colonies picked**. The SISPA method was performed for enterobacteriophage M13 (6407 bp), Newcastle disease virus Lasota (15,186 bp) and enterobacteriophage lambda (48502 bp). One, two or three 96-well blocks of clones were sequenced, trimmed and assembled. The sum of the total lengths of edited contigs for each condition was calculated as percent of the total reference genome length.

Figure 4A shows an analysis of sequence coverage for the viruses examined in this study. On average, four contigs were generated per experiment, ranging in size from 248 nt to 4495 nt with a median contig size of 1395 nt. The contigs had high sequence redundancy, with a median depth of coverage of 14.33, varying from 11.18 for turkey astrovirus (TA) to a high of 40.29 for MS2.

One parameter that is taken into consideration when designing an efficient protocol for construction of a sequence library is the number of independent colonies needed to obtain sequence coverage of a given reference genome. Experiments were conducted using M13 (a 6 kb genome), NDV (a 15 kb genome), and lambda phage (48.5 kb) to compare the level of coverage obtained by bidirectional sequencing of 96, 192, and 288 clones (Figure 4B). For M13, 94% genome coverage was achieved from sequencing one 96 well block of clones, and 97% genome coverage was obtained from two 96 well blocks. For NDV, 89.7%, 97.4% and 97.7% sequence coverage were obtained from one, two or three 96 well blocks respectively. In contrast to M13 and NDV, the coverage for lambda was 26.7%, 42.9%, and 52.4% after one two and three 96 well blocks were sequenced

The efficiency of the SISPA method as a tool for obtaining full genome coverage was analyzed using the Lander and Waterman model [17], which estimates the number of gaps present as a function of sequence number and genome size. Table 2 compares the expected coverage and redundancy (depth of coverage) as predicted by the Lander-Waterman model with the observed genome coverage and redundance. With the exception of lambda phage, observed coverage and redundancy approach expected coverage and redundancy.

**Table 2:** Lander-Waterman analysizs of viral genome coverage.

Virus Name	Total Sequences	Observed Coverage	Expected Coverage	Observed Redundancy	Expected Redundancy
WHV	121	0.84	1.00	14.86	18.45
MS2	283	0.93	1.00	40.29	40.20
M13	232	0.90	1.00	21.26	18.36
HRV 16	195	0.90	1.00	14.33	13.88
TA	148	0.93	1.00	11.29	10.22
NDV	349	0.97	1.00	13.72	11.65
Lambda	281	0.52	0.95	3.72	2.92

Observed coverage and redundancy was compared with the expected coverage and redundancy as predicted by the Lander-Waterman model for the total number of sequences in each assembly.

However when taking into account the scaled difference, as described by Wendl [18], we see a dramatically increased "shortfall" between actual and expected coverage as more clones are sequenced. For example, in the case of NDV which has a genome size of 15 Kb, the scaled difference *D *between the expected coverage and the observed coverage (see equation description in methods section) at the different levels of sequence redundancy is 48.3 for the sequencing of a plate of 96 clones, 464.4 for two plates and 5477.4 for three plates.

The SISPA method works efficiently on viruses purified from a number of sources and by several methods. Enterobacteriophages M13, MS2, and lambda were isolated from bacterial growth media and plasma after concentration by density gradient centrifugation. Woodchuck hepatitis virus was purified from plasma by cesium chloride gradient centrifugation. Human rhinovirus 16, purchased as a cell culture supernatant from ATCC, was subjected to a low speed spin to remove cellular debris. Turkey astrovirus was isolated from fecal material collected from turkey poults showing clinical signs of diarrhea. The intestinal fecal content was diluted in PBS and centrifuged at 14,000 K before filtration and nuclease treatment. Newcastle disease virus RNA was purified from allantoic fluids derived from inoculated eggs.

**Figure 5:**
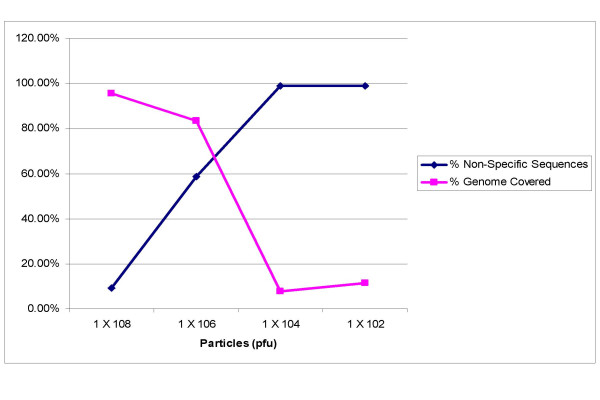
**Relationship between initial virus particle number, genome coverage and percent non-specific sequences generated by SISPA**. MS2 viruses were diluted to 10^8^, 10^6^, 10^4^, and 10^2 ^particles per SISPA DNAse I reaction. The sum of the total lengths of edited contigs for each dilution was calculated as percent of the total reference genome length. Non-specific sequences were determined as those sequences which did not match reference genome with a cutoff value less than 10^-25^.

To determine the number of viral particles necessary to generate full genome sequences, we conducted dilution series with viruses whose titer was determined by plaque assays. The results of these experiments demonstrate that the SISPA method is very efficient as a genome sequencing method for samples with greater than 10^6 ^viral particles per RT-PCR reaction (Figure 5). Below 10^6 ^particles, the specific viral signal is overwhelmed by competition with non-specific or host sequences and is rarely detected from sequencing two blocks (192) of colonies.

### Resolution of 3' and 5' ends

Our initial results indicated low sequence coverage at the 3' and 5' ends of most viral genomes. In order to address this problem in viruses with polyA tails the FR40RV-T primer (Figure 2) is added to the RT reaction. This increases the number of cDNAs produced at the 3' end of the genome, and results in a much greater depth of coverage at the 3' end. The polyT containing primer is added to the RT reaction at a concentration 200 fold lower than the random primer in order to reduce competition with the random primer.

We used human rhinoviruses to develop the methodology for improving the coverage of the 5' end. We took advantage of a conserved region from nucleotide 1 to nucleotide 10 in the 5' untranslated region. The conserved primer was used in the Klenow step of the SISPA protocol to enrich for the presence of amplicons from the 5' end. When used in combination with the 3' primer, we have been able to obtain full rhinovirus genome coverage in a 192 clone experiment (data not shown).

### Contaminant sequences

One inherent difficulty of a method that relies on a random reverse transcription and PCR to generate amplicons for sequencing is the likelihood of detecting contaminant sequences as well as sequences of interest. Although filtration and nuclease treatment does reduce the presence of nucleic acids from whole cells and host chromosomes, contaminating RNA species will inevitably remain and thus be amplified (Table 3).

**Table 3:** Specific sequences and contaminants in turkey astrovirus and human rhinovirus 16 assemblies.

**TURKEY ASTROVIRUS**		
**Contig Length**	**Sequence reads**	**Best Hit**

689	2	Avian
501	2	None
423	2	None
518	2	None
259	2	None
1267	28	Turkey astrovirus
2785	67	Turkey astrovirus
1692	43	Turkey astrovirus
**Total Sequences**	148	
**Percent Specific**	93.24%	
**Genome coverage**	90.84%	
		
**HUMAN RHINOVIRUS 16**		

**Contig Length**	**Sequence Reads**	**Best Hit**

265	8	Mammalian
537	2	Mammalian
981	27	Mammalian
1091	7	Bacterial
1297	10	Bacterial
553	1	None
385	1	None
342	3	None
815	32	None
909	18	None
676	8	None
487	9	None
4823	105	Human Rhinovirus 16
1685	90	Human Rhinovirus 16
**Total Sequences**	321	
**Percent Specific**	60.75%	
**Genome coverage**	90.23%	

Sequences were analyzed against a non redundant database using a blastn algorithm. Viral specific sequences were identified as matching the reference genome with a blastn cut off below 10^-25^. Non-specific (non-viral contaminant) sequences were identified if they had a cut off value below 10^-10^, while None means that no blastn results were found below the 10^-10 ^cut off value.

To determine the presence of contaminant sequences in the clone population, all generated sequences were subjected to a blastn search against the NCBI (non-redundant) database. A cutoff e value of 10^-25 ^was used to identify viral sequences which matched the reference genome. Non-specific sequences (i.e., those that did not match the input viral isolate) were identified as mammalian, avian, bacterial, etc., if their best hit was below a cut off value of 10 ^-10^. If no blast results were found below the 10 ^-10 ^cut off value the sequences were not given a specific designation. In experiments resulting in nearly complete genome sequences, contaminant sequences ranged from 3–40%. The nature of the contaminant sequence depended on the initial viral host and included mammalian, avian, bacterial, fungal, viral and unknown sequences. In the case of rhinoviruses, which were purified from HeLa cell culture, the majority of contaminant was of derived from human or mycobacterial nucleic acids. Newcastle (NDV) and astrovirus (TA) which were purified from chicken egg allantoic fluid and turkey feces, respectively, were contaminated primarily with nucleic acids of avian origin. Table 3 shows the results of blast analyses of two samples, TA and HRV16.

## Conclusion

The work presented here demonstrates the utility of the random genome sequencing method for the generation of viral sequence from positive strand ssRNA (Human Rhinovirus, Turkey astrovirus) and negative strand ssRNA viruses (Newcastle disease virus), ssDNA (enterobacteriphage M13) and dsDNA viruses (woodchuck hepatitis virus and lambda phage). In addition, using the DNase I-SISPA technique we were able to amplify sufficient target material for sequencing from various sources, including cell culture isolates and field isolates which have not been purified by ultracentrifugation. Although ultracentrifugation is an efficient procedure to purify viruses, it is not practical for processing samples of relatively low viral titer in a small volume or high throughput processing of viral samples for genomic sequencing.

Genome coverage and redundancy for viral samples from 3–15 kb approach the ideal values as predicted by the Lander-Waterman model [17]. However, as the sequence number increases, the efficiency of the method as measured by the scaled difference [18] decreases dramatically. Thus, while the number of gaps declines as more clones are sequenced, the efficiency is reduced (i.e. there is more 'loss'. Remaining gaps and areas of 1× coverage may be due to regions of secondary structure, hydrolysis of the RNA template or cloning bias. Additionally, AT rich regions may inhibit the annealing of random primers during the RT, Klenow or PCR step. We routinely pick a total of 192 clones (or two 96 well blocks) per viral sample for bidirectional sequencing as this represents the most affordable sequence coverage to efficiency ratio. While significant coverage is obtained from a single experiment, final genome assembly requires varying levels of targeted RT-PCRs to close the genome (Figure 3).

The 3' end of the virus generally has the lowest coverage in any use of this protocol. In theory, given the directionality of the reverse transcriptase (3' to 5') and assuming an equal distribution of binding sites for the random primer, the 5' end of any viral genome will get higher depth of sequence coverage than the 3' end. We have found that addition of a tagged oligo dT primer significantly reduces this problem for viruses with polyA tails (most positive ssRNA viruses), but this remains a limitation for other virus genome types. The 5' end of most viruses has also proved difficult to complete and we have found that the addition to the RT reaction of degenerate oligos based on conserved 5' sequences can increase coverage. However we have not been able to develop a universally applicable method for obtaining complete 5' coverage. We strongly anticipate that specific adaptations of the SISPA method to conserved regions of different viruses will demonstrate its versatility in a wide range of viral genome sequencing initiatives.

Limitations to the method include the need for samples containing a minimum of 10^6 ^particles (in the original 1 ml or 0.2 ml samples). Moreover because the capsid structure renders the viral genomes nuclease-resistant, this protocol requires encapsidated viral genomes to allow the removal of most extra-viral contaminants. The viral nucleic acids in samples whose capsid structures have been disrupted cannot be separated from contaminants, and therefore cannot be efficiently amplified by SISPA. In the experiments discussed in this paper DNAse I was used to reduce host contaminant. For samples with high levels of host nucleic acid contaminant, we have used 5 μg of RNAse A to treat 500 μl of filtered virus for 1 hr. We have found that RNAse A treatment eliminates the majority of host RNA derived sequence contaminant in these cases.

The SISPA method is particularly useful for obtaining genome sequence from RNA viruses. Because most sequencing methods for RNA viruses depend on RT-PCR with primers designed from pre-existing sequence data, the utility of this protocol is particularly evident for highly variable or degenerate viral families or for viruses with little available sequence information. In addition, the SISPA method will be useful for uncharacterized viruses as no prior sequence information is required.

## Methods

### Preparation of viral nucleic acids

Viral RNA and DNA was prepared following the guidelines provided by [1,2] with some modifications. Culture supernatants purchased from the American Tissue Culture Collection (**ATCC**) and other virus-containing biological samples were prepared for viral RNA and DNA extraction. Each biological sample was first spun to remove cellular debris and processed through a 0.22 μM filter to enrich viral particles in the flow-through while retaining bacterial and other large cells in the filter. When necessary, viral particles were concentrated by ultracentrifugation at 149, 000 × g (45, 000 RPM in a 70.ti Beckman rotor) and the pellet was resuspended in 200 μl of 2% Normal Human Serum prepared in sterile water. To eliminate residual nucleic acid contaminants in the filtrate, 100 units of DNAse I and/or 10 μg/ml RNAse A was added to the viral resuspension and was incubated at 37°C for 1 hour. RNA and/or DNA was then isolated from the sample. For viral RNA preparation, the Trizol-LS reagent (Invitrogen) was used according to the manufacturer's instructions. The RNA pellet was resuspended in 20 μl of nuclease-free water. To prepare viral DNA, the QIAmp DNA Preparation kit (Qiagen, Cat. # 51104) was used as recommended and the DNA was eluted with 50 μl of nuclease-free water.

### Construction of a library of random PCR fragments and sequencing

The extracted RNA was processed for random reverse transcription as previously described [1,2] using the FR26RV-N primer (5' GCC GGA GCT CTG CAG ATA TCN NNN NN 3') at a concentration of 1 μM. In addition, FR40RV-T (5' GCC GGA GCT CTG CAG ATA TC (T)_20 _3') was added at a concentration of 5 nM to specifically amplify the 3' end of positive strand viruses. After the first cDNA synthesis, the double stranded cDNA was synthesized by Klenow reaction the presence of random primers. In order to amplify 5' ends of rhinoviruses the following primer was added to the Klenow reaction at a concentration of 10–20 nM (5'GCC GGA GCT CTG CAG ATA TC TTA AAA CTG G 3'). PCR amplification used high fidelity Taq Gold DNA polymerase (ABI) with the FR20RV primer (5' GCC GGA GCT CTG CAG ATA TC 3'). PCR amplicons were A-tailed with dATP and 5 units of low fidelity DNA polymerase (Invitrogen) at 72°C for 30 minutes. A-tailed PCR amplicons were analyzed in a 1% agarose gel and fragments between 500 and 1000 nt were gel purified. Amplicons were ligated *en masse *into the Topo TA cloning vector (Invitrogen) and transformed into competent one shot Topo top 10 bacterial cells (Invitrogen). For DNA viruses, the purified viral DNA was denatured and complementary strands synthesized by Klenow reaction as indicated for ds-cDNA from first strand cDNA. Clones were plated on LB/Amp/XGal agar, and individual colonies were picked for sequencing. The clones were sequenced bidirectionally using the M13 primers from the topo TA vector. We routinely sequenced a total of 192 or more per library. Sequencing reactions were performed at the Joint Technology Center (an affiliate of the J Craig Venter Institute: JCVI) on an ABI 3730 xl sequencing system using Big Dye Terminator chemistry (Applied Biosystems).

### Analysis of genome coverage

In the Lander and Waterman analysis of genome coverage [17] G = Size (bp) of Reference Genome, L = Sequence Length (bp) and N = # sequences; Redundancy represents the depth of sequence coverage and Coverage represents the fraction of genome covered by sequence data.

The Ideal Redundancy (R) = LN/G and the Ideal Coverage = 1-e^-R ^[17]

Observed Coverage = sum of the length of all contigs/G. Observed Redundancy = the average of total sequence length (length of all sequence reads in a contig including gaps)/contig Length. Both Observed coverage and Observed Redundancy are experimentally derived values. The average sequence read size for the experiments described was 507.83 +/- 47.16 bp.

The loss of coverage due to various biases is represented as the difference between the ideal coverage and the actual coverage. To allow quantitative comparison, this 'shortfall' difference is scaled by the standard deviation of the coverage probability distribution as given by Wendl [18]. Following Wendl, we use the moments of the vacancy (which is the complement of the coverage) to calculate the standard deviation.

*E*⟨*V*⟩ = (1 - *α*)^*N*^

Where *α *is the ratio of the read length and the genome length and *N *is the number of reads.

The second moment is given as:

*E*⟨*V*^2^⟩ = *exp*(-2*ρ*) + 2/*N*·*exp*(-*ρ*)

Where *ρ*, the redundancy, is define to be equal to *Nα*

The expression for the variance is then:

*σ*^2 ^= (1 - *α*)^2*N *^- *exp*(-2*ρ*) + 2/*N*·*exp*(-*ρ*))

The standard deviation is then:

$$S=\sqrt{{\left(1-\alpha \right)}^{2N}-(exp(-2\rho )+2/N\cdot exp(-\rho ))}$$

The ideal coverage is given by:

*E*⟨*C*⟩ = 1 - *exp*(-*ρ*)

Using the standard deviation for the vacancy in place of that for the coverage, the correctly-scaled difference *D *between ideal coverage and the actual coverage *A *is:

*D *= (*E*⟨*C*⟩ - *A*)/*S*

Note that for large *N *the mean vacancy converges to *exp*(-*ρ*) allowing the following simplified approximation of *S*:

$$S=\sqrt{2/N\cdot exp(-\rho )}$$

### Assembly of viral genomes

Sequence reads were trimmed to remove amplicon primer sequence as well as low quality sequence, and assembled. A small genome assembler called Elvira (Executive for Large-scale Viral Assembly), based on the open-source Minimus assembler, was developed to automate these tasks. For some figures images were generated using the SeqMan II sequence analysis software, DNASTAR Inc. 1989–2002.

### Reference genomes

The following complete genomes were used as reference genomes for the viruses discussed in this study: Woodchuck hepatitis virus (AY628095); Enterobacteriophage MS2 (NC_001417); Enterobacteriophage M13 (NC_003287); Human Rhinovirus 16 (L24917); Turkey Astrovirus (NC_002470);

Newcastle disease virus LaSota (AY845400) Enterobacteria phage lambda (NC_001416)

## List of abbreviations

SISPA: Sequence-independent single primer amplification; rPCR: Random polymerase chain reaction; RT: Reverse transcription; NDV: Newcastle disease virus LaSota; TA: Turkey astrovirus 2; HRV: Human rhinovirus.

## Competing interests

The author(s) declares that there are no competing interests.

## Authors' contributions

AD participated in drafting the article, experimental design, and data analysis and carried out molecular studies. JD participated in data analysis. RH, RK, JF and NS participated in experimental design and carried out molecular studies. CA, XZ and NG provided materials used in the study. EG participated in experimental planning and drafting the manuscript. DS conceived of and coordinated the study, and participated in data analysis and drafting the manuscript.
